# *Lamiaceae* Essential Oils, Phytochemical Profile, Antioxidant, and Biological Activities

**DOI:** 10.1155/2021/6748052

**Published:** 2021-12-14

**Authors:** Luiz Renan Ramos da Silva, Oberdan Oliveira Ferreira, Jorddy Nevez Cruz, Celeste de Jesus Pereira Franco, Tainá Oliveira dos Anjos, Marcia Moraes Cascaes, Wanessa Almeida da Costa, Eloisa Helena de Aguiar Andrade, Mozaniel Santana de Oliveira

**Affiliations:** ^1^Programa de Pós-Graduação Em Ciências Biológicas-Botânica Tropical, Universidade Federal Rural da Amazônia and Museu Paraense Emílio Goeldi, Av. Perimetral, 1901. Terra Firme, Belém 66075-900, Pará, Brazil; ^2^Adolpho Ducke Laboratory-Botany Coordination, Museu Paraense Emílio Goeldi, Av. Perimetral, 1901, Terra Firme, Belém 66077-830, Pará, Brazil; ^3^Postgraduate Program in Biodiversity and Biotechnology-Bionorte Network, Universidade Federal do Pará-Rua Augusto Corrêa S/N, Guamá, Belém 66075-900, Pará, Brazil; ^4^Postgraduate Program in Chemistry–Universidade Federal do Pará-Rua Augusto Corrêa S/N, Guamá, Belém 66075-900, Pará, Brazil; ^5^Universidade Federal do Pará-Rua Augusto Corrêa S/N, Guamá, Belém 66075-900, Pará, Brazil

## Abstract

Medicinal and aromatic plants present important active compounds that have potential for use in food, pharmaceutical, and agriculture industries. In this sense, the present work aimed to conduct a literature review on the potential applications of essential oils from *Lamiaceae* species. Antioxidant, anti-inflammatory, and antimicrobial activities were evaluated. The importance of this study is demonstrated as a way to theoretically provide information on the use of different plants belonging to the *Lamiaceae* family, especially with regard to the physical, chemical, and biological properties of its essential oils.

## 1. Introduction

Several studies have shown that plants have bioactive compounds, such as terpenoids, alkaloids, glycosides, phytohormones, phenolic, and phenylpropanoids, that assist in the development of phytotherapeutic; in addition, natural products can be a viable alternative for the development of new drugs to control microorganisms resistant to traditional antibiotics [1–6]. *Lamiaceae* family has several species of aromatic plants that are applied in traditional medicine and in the pharmaceutical and food industries because of their biological properties [7]. They are used as stimulant for blood circulation and digestion, to strengthen the central nervous system, and as expectorant, antispasmodic, antiseptic [8], diuretic, carminative, and tonic [9]. The most popular plants in this family are oregano, rosemary, thyme, and sage [7].

The biological applications of the *Lamiaceae* are mainly related to its essential oils, which have various activities such as antioxidant, antitumor, anti-inflammatory, antiviral, analgesic, antitussive, antiasthmatic, antipyretic [9], antimicrobial, antiemetic, antifungal [10], insecticidal (against *Aedes aegypti*) [11], antidiabetic, antihypertensive [12], antipruritic, decongestant [13], antinociceptive [14], carminative, antirheumatic, antidepressant, neuroprotective, cholinergic [15], sedative, antiseptic, antiparasitic, anthelmintic, immunoregulatory [16], antiallergic [17], antiangiogenic, anti-hepatotoxic [18], anticancer [19], and others.

According to Pires et al. [20], medicinal plants began to be used both in traditional medicine (*in natura*) and in vegetal products, such as essential oils, enhancing the investigations of plant species and, consequently, their natural pharmacological agents, considering the different perspectives of rural and urban areas.

According to data from the WHO, more than 70% of the population uses herbal medicines as the main form of medication to treat diseases [21]. This growing interest for less industrialized products with functional ingredients stimulated the use of essential oils in several industrial sectors (food, cosmetics, hygiene, and agriculture), which are applied in product conservation and control of microorganisms [22–24]. The advantage of volatile oils over synthetic preservatives lies in the lower development of toxic side products and economic viability [25, 26].

The essential oils (EOs) are aromatic and volatile substances found in different plant parts (leaves, flowers, seeds, roots, and fruits) [26]. Also, according to the de Oliveira et al., [27], they are extracted by steam distillation, dry distillation, mechanical extraction, or other processes such as supercritical CO_2_ extraction. Essential oils are capable of undergoing physical processes which do not significantly alter their chemical compositions. The extraction methods vary according to the species, the plant part used, and the way the raw material is presented: fresh, partially dehydrated, or dried [28–30].

In general, EOs are formed by base elements (oxygen, hydrogen, and carbon), which generate aldehydes, esters, phenols, ketones, alcohols, organic acids, and substances with nitrogen/sulfur, hydrocarbons, and terpenes [31]. These functional groups are responsible for the properties of the oils. Those commonly found come from terpenoids and phenylterpenoids, with monoterpenes being the most frequent [32–34].

For the plant, EOs represent an adaptive advantage, being able to function as an attractant for pollination and as a natural defense against predator attacks [35]. The chemical composition of essential oils can vary within the same species because conditions such as cultivation site, collection method, environmental factors, and material storage can interfere in the production of metabolites [36].

The family to which plants belong can be important to make inferences about the composition of aromatic oils. For instance, *Rutaceae* family presents citrus species; *Myrtaceae* has as representative the eucalyptus; and *Oleaceae*, the jasmine [37–39]. *Lamiaceae* is one of the plant families that presents great interest regarding obtaining essential oils, which will be described in the next topics.

Thus, this paper is organized, besides introduction and final considerations, in three parts: (1) biological presentation and identification of *Lamiaceae* species; (2) chemical structures of biosynthesized molecules present in its EOs; and (3) different properties of these species.

## 2. *Lamiaceae* Family

The plant family *Lamiaceae*, formerly called Labiatae, for its flowers are characterized by a bilabiate corolla [40], *Lamiaceae* presents more than 7000 species that are grouped in about 240 genera; in Brazil, it is distributed in approximately 524 species belonging to 52 genera [41], and some of these species present aromatic properties, which confers great economic relevance to the *Lamiaceae* [42], being applied in cosmetics and herbal medicines. As examples of genera with such properties, *Mentha, Ocimum, Salvia, Clerodendrum*, and *Plectranthus* stand out.

*Lamiaceae* species are widely distributed around the globe, with various heights and habitats and greater abundance in the Mediterranean region [43]. They prefer hot areas; however, they can also be found in regions with low temperatures [41, 44]. In Brazil, *Lamiaceae* species are distributed throughout the country, with higher incidence in south, north, and northeast regions, comprising the Atlantic Forest, Amazon, and especially the Brazilian Cerrado [45–48].

The species of the *Lamiaceae* family have diversified morphological characteristics and may be herbs, herbaceous plants, shrubs, or tree species [41]. Nowadays, this is one of the biggest botanical families with flowers of different sizes, with warm and showy colors depending on the species. They are bisexual, with well-defined floral parts, apparent sepals and petals, inflorescence, and bilateral symmetry (zygomorphs), and the corolla tube is divided into two distinct parts, providing a “lip” shape, which is the main characteristic of the Labiatae family. Their leaves are normally simple, and their fruits are dry and multiple that become separated when ripe (schizocarpic fruits) [40, 49, 50].

This family presents many species rich in flavonoids and terpenes, with diterpenoids being the most abundant [51]. They are also rich in other substances that in addition to providing medicinal use have also assisted in taxonomic classifications [52]. Among the spices with aromatic properties, the six best-known vernacular names are thyme, basil, oregano, rosemary, sage, and lemon balm [16]. This variety of bioactive compounds confers *Lamiaceae* properties such as antioxidant, insecticidal, fungicidal, and bactericidal [53], which can result in an aggregation of potential economic and pharmacological value.

## 3. *Lamiaceae* Species Rich in Essential Oils

Species of the *Lamiaceae* family produce large amounts of secondary metabolites, including the compounds present in essential oils in plants with biological activities and therapeutic potential [41, 44]. Some examples include species *B. officinalis, G. hederacea, H. pectinata. Lavandula. Lamium, M. officinalis, Mentha, M. vulgare, Origanum, Ocimum, R. officinalis, Salvia, S. hortensis, S. lavandulifolia, S. lateriflora, Sideritis, Teucrium, Thymus,* and *Ziziphora tenuior* [54].

The genus *Plectranthus* is considered one of the richest in species diversity and essential oils, with monoterpenes and sesquiterpenes as the main constituents [55]. According to Crevelin et al. [56], the essential oils of *Plectranthus neochilus* and *Plectranthus barbatus* have antimicrobial effects against *Streptococcus mutans*. Besides antibacterial activity, *Plectranthus* also has antifungal action on *Rhizopus stolonifer* [57] and showed *in vitro* antischistosomal activity attributed to boldo essential oil, which exterminated 100% of *Schistosoma mansoni* adult worms [58]. It also caused reduction in female eggs of B-type *Bemisia tabaci* in tomatoes [59, 60]. *Plectranthus amboinicus* exhibited anti-inflammatory and good digestion activities, as well [61].

Among the herbaceous plants of the *Lamiaceae* family, the genus *Ocimum* is the most important due to its application in several areas [62], such as folk medicine, cooking, plant marketing, and perfumery industry [63]. Approximately 30 species compose this genus [63]. Among them, some are *Ocimum gratissimum, O. basilicum L., O. micranthum,* and *O. campechianum*. The extracts are applied in traditional medicine to treat rheumatism, epilepsy, some mental conditions, and respiratory tract infections [64–66]. Studies also have verified fungicidal, nematicidal, and larvicidal properties [67–70].

Additionally, the antifungal action of essential oils from *Lamiaceae* species has been used to improve food preservation. Isolated essential oils derived from thyme and oregano (thymol), clove (eugenol), and mint (menthol) were tested in strawberry preservation [71]. As a result, the treatment reduced strawberry degradation when compared with the control sample. Thymol oil showed better results, with a decay of 0% on day 1 to 20 on day 14, with better results than the control sample, and the authors concluded that in addition to antimicrobial activity, treatment with essential oils also conferred antioxidant protection.

The essential oil of *O. gratissimum* L. was able to inhibit the growth of species such as *Klebsiella* sp., *Pseudomonas aeruginosa*, *Escherichia coli*, *Staphylococcus aureus,* and *Salmonella enteritidis*, even when used at different concentrations [72]. Pereira et al. [73], evaluated the antibacterial activity of *O. gratissimum L*., *Cymbopogon citratus* (DC) Stapf., and *Salvia officinalis* L. on microorganisms isolated from urinary tract infections. *Salvia officinalis* L. showed the best results, inhibiting the growth of more than 75% of all microorganisms evaluated. Species such as *Salvia santolinifolia L, Salvia hydrangea L, Salvia mirzayanii L, Salvia triloba L, Salvia repens L,* and *Salvia runcinata L.* also stand out.

The genus *Hyptis* (*Hyptis ovalifolia* Benth, *Hyptis suaveolens* L, and *Hyptis pectinata* L.) is predominant in the semiarid region of Northeastern Brazil, with prevalence of herbs but also with shrubby representatives and small trees [74]. Its EO has antiseptic, insecticidal, and fungicidal activities in addition to treating gastrointestinal infections and muscle pain [72, 75]. In addition, the genus *Perilla*, whose main representative is *Perilla frutescens* L., has insecticidal activity, which is given by the isolated compound [76].

Genus *Mentha*, popularly known as mint, has menthol terpenes as main constituents of its essential oil. The greater yield is found in its leaves, presenting a considerable economic potential for food and pharmacological purposes [77, 78]. This genus has a small diversity of plants all over the globe, containing only 25 species [79]. The biological activities presented by these *Lamiaceae* species are varied, e.g., antihypertensive, antioxidant, antimicrobial, antiallergic, biopesticidal, antitumor, anticancer, anti-inflammatory, and antiviral [80], which may be associated with the presence of compounds such as menthol, menthone, 1,8-cineole, carvone, limonene, *ß*-caryophyllene, and pulegone, among others [81].

Research on the genus *Satureja* L. reports that it is distributed in 30 species around the globe, and that it has beneficial properties for human health, such as in the treatment of pain caused by oxidative stress. Therefore, the essential oils from leaves and stems of *Satureja spicigera* L*, Satureja cuneifólia* L, and *Satureja hortensis* L. have compounds that work as antioxidants [82, 83].

Genus *Thymus* presents about 200 species. Most of them have antibacterial action due to the high content of phenolic compounds. *T. caespititius, T. camphoratus*, *T. pectinatus* Fihch,* T. mastichina* L., and *Thymus vulgaris* L. [84] are great examples, whereas *T. numidicus* and *T. fontanesii* have bactericidal activity [85].

## 4. Chemical Composition of *Lamiaceae* Essential Oils

Essential oils are volatile, lipophilic, and odoriferous substances produced by the secondary metabolism of plants. Due to their aromatic properties and chemical composition, they are used in cosmetics and folk medicine, with antiseptic, antifungal, and insecticidal actions [7].

In general, the biological activities that essential oils present are observed by the major substances present in their chemical composition. Their bioactivity is shown synergistically or by the isolated substances [86, 87].

In addition, the chemical composition of an essential oil can vary depending on the species, seasonality, circadian rhythms, plant age, and geographic location [36, 88]. As an example, the chemical profile of *Hesperozygis myrtoides* essential oil, which is a subshrub native to Cerrado and Atlantic Forest of Brazil, depends on altitude [89].

Essential oils are characterized by two or three major constituents. For instance, *Mentha arvensis* L. presented as major compounds menthol (86.1%), menthone (4.3%), and isomenthone (3.7%) [90]; *O. gratissimum* L. showed as major constituents 1,8-cineole (30.04%), eugenol (27.58%), and terpineol-4 (14.45%) [91]; *Origanum vulgare* L. presented 4-terpineol (18.4%), sabinene hydrate (15.6%), and thymol (13.6%) [92]; and in the species *Plectranthus ornatus* Codd, the major compounds identified were *α*-thujene (12.7−32.7%), *α*-pinene (5.5−23%), sabinene (7.51−17.8%), *β*-pinene (3.5−11.6%), 1-octen3-ol (0.6−11.1%), 3-carene (0.84−5.6%), (E)-*β*-ocimene (1.5−8.4%), *α*-terpinyl acetate (1.3−13.2%), *β*-caryophyllene (3.9−13.6%), and germacrene D (0.3−18.5%) [93].

Giatropoulos et al. [94] evaluated 12 species of different plants of the *Lamiaceae* family and found high insecticidal action in the essential oils of *T. vulgaris* and *O. vulgare*. Such properties can be attributed to the high toxicity of its major constituents such as thymol (75.6%), carvacrol (74.08%), and p-cymene (7.9%). The considerable toxicity of *Satureja thymbra* essential oil is also observed probably due to its major constituents carvacrol (32.4%) and *γ*-terpinene (32.4%).

**Figure 1:**
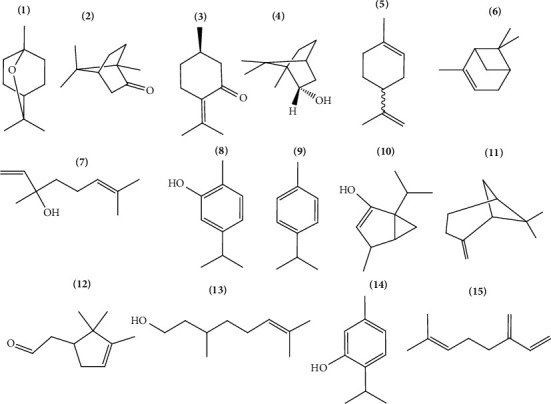
Monoterpenes: (1) = 1,8-cineole, (2) = camphor, (3) = pulegone, (4) = borneol, (5) = limonene, (6) = *α*-pinene, (7) = linalool, (8) = carvacrol, (9) = p-cymene, (10) = thujanol, (11) = *ß*-pinene, (12) = *α*-campholenal, (13) = citronellol, (14) = thymol, and (15) = *ß*-myrcene.

**Figure 2:**
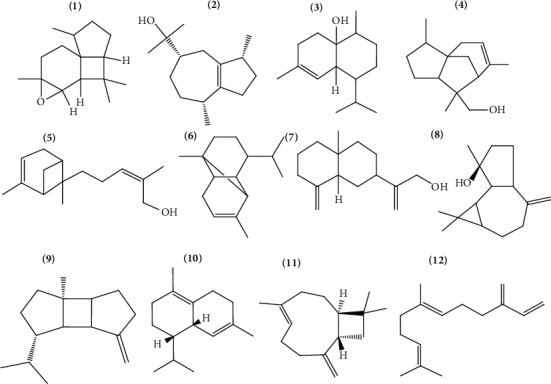
Sesquiterpenes: (1) = Italicene epoxide, (2) = guaiol, (3) = 1,10-di-epi-cubenol, (4) = 8-cedren-13-ol, (5) =  (Z)-*α*-trans-bergamotol, (6) = *α*-copaene, (7) = *ß*-costol, (8) = spathulenol, (9) = ß-bourbonene, (10) = *δ*-cadinene, (11) = ß-caryophyllene, and (12) = ß-farnesene.

Thus, the number of studies that seek applicability of the compounds present in *Lamiaceae* essential oils has increased since they have natural origin and present advantages when compared with synthetic substances [7].  Table 1 lists species rich in essential oils and their main constituents. Figures 1 and 2 show the main monoterpenes and sesquiterpenes identified in *Lamiaceae* essential oils.

## 5. Antioxidant Activity

Antioxidants are substances capable of retarding or preventing lipid oxidation caused by excessive oxygen radicals due to environmental factors or pathogens [110, 111]. Such compounds, which can be natural or synthetic, have great importance in the food industry because they are used as preservatives in several products, delaying or preventing deterioration caused by the action of oxygen. Besides this, antioxidants have great relevance in biochemical and medical fields because they are able to neutralize the harmful effects of oxidation in animal tissues [112].

In recent years, there has been an increasing search for natural products with antioxidant properties due to the toxic side problems that synthetic products may cause [110]. Aromatic and medicinal plants are considered natural sources of antioxidant substances since their secondary metabolites act by inhibiting the formation of free radicals [113]. The aromatic and medicinal species of the *Lamiaceae* family have been constantly studied regarding their antioxidant activities, as shown in Table 2.

There are several techniques that determine the antioxidant capacity of essential oils and their components, among them are FRAP (ferric reducing antioxidant power), CUPRAC (cupric ion reducing antioxidant capacity), ABTS (2,20-azino-bis (3-ethylbenzothiazoline-6-sulfonic acid)), DPPH (2,2-diphenyl-1-picrylhydrazyl), quantification of products formed during lipid peroxidation (TBARS, LDL oxidation, and co-oxidation of *ß*-carotene), and other methods [131].

The chemical composition and antioxidant activity of *O. vulgare* essential oil were studied by Morshedloo et al. [124]. After being analyzed by the DPPH method, all oils presented antioxidant activity, which was correlated with the high concentration of carvacrol. The essential oil from *O. vulgare* flowers showed the highest capacity to eliminate DPPH radicals (EC_50_ = 0.68 mL/mL), while the stem oil showed the lowest capacity (EC_50_ = 1.82 mL/mL). Regarding phenological stages, flowering showed the highest antioxidant activity (EC_50_ = 0.86 mL/mL) [123]. The authors also pointed out the strong antioxidant action of the essential oil from *Origanum vulgare* aerial parts using ABTS radical scavenging technique with IC_50_ = 14.00 *μ*g/mL.

Besides *O. vulgare*, other species of the genus *Origanum* are reported in the literature to possess antioxidant activity. The essential oil of *O. dictamnus* flowers showed antioxidant activity by the DPPH method (IC_50_ = 0.0459 ± 0.0042% (v/v)) that was attributed to its main compound, carvacrol [121]. The oils from aerial parts of *O. floribundum* were studied by Hadjadj et al. [122] regarding their antioxidant potential by DPPH and ABTS assays. They presented better antioxidant activity by the ABTS method (33.6–95.5 *µ*g/mL).

Zorzetto et al. [114] evaluated the antioxidant activity of *Cedronella canariensis* aerial parts using three methodologies (DPPH, ABTS, and FRAP). The authors demonstrated that the essential oil showed better antioxidant activity against the ABTS radical with IC_50_ = 10.5 mg/mL, which was about 20 times lower than Trolox. Although the DPPH method is similar to ABTS, *C. canariensis* oil presented low antioxidant activity (IC_50_ = 615.5 mg/mL), about 500 times lower than Trolox. In addition, EOs of *O. basilicum* were shown to possess antioxidant activity by DPPH (IC_50_ = 0.21–4.04 mg/mL) and *ß*-carotene (bleaching content = 23.8–85.3%) [120].

*Rosmarinus officinalis* is known to possess several biological properties. Aerial parts of this species were collected in southeastern Anatolia (Turkey), and its essential oil showed antioxidant activity by DPPH and TBARS techniques with IC_50_ = 10.08 ± 0.15 *µ*g/mL and 1.76 ± 0.02 *µ*g/mL, respectively, which may be related to polyphenols and phenylpropanoids found in the oil [125]. Moghadam [126] showed that the essential oil of *R. officinalis* collected in Kermanshah (Iran) presented antioxidant activity, by DPPH assay (13.00 ± 0.51 *μ*g/mL), which was related to the presence of camphene and 1,8-cineole. Table 2 shows the relationship between *Lamiaceae* species and their antioxidant potential.

## 6. Anti-Inflammatory Activity

Inflammation is a sequential process produced by various biological stimuli, physical injuries, infectious agents, and antigen-antibody type interactions. Within the inflammatory process, there are reactive oxygen species (ROS) responses, which include superoxide anions, hydroxyl radicals, and hydrogen peroxides. These are released by activated macrophages, neutrophils, and dendritic cells [132].

The inflammatory process and its chain of development have presented relevance, and in this sense, intending to restrain this aggressive action on the organism, search for new anti-inflammatory agents, mainly of vegetable origin, is necessary [133]. It is worth emphasizing that within this branch, species such as *Hyptis spicigera,* which is used in folk medicine, have anti-inflammatory properties [134].

Essential oils from *O. basilicum* and *O. gratissimum* were obtained by hydrodistillation and hexane extraction. *O. basilicum* EO extracted by hydrodistillation presented eucalyptol and eugenol acetate, and the solvent extract presented 2-methylbenzyl and eugenol acetate. Regarding *O. gratissimum* EO, linalool, 1-terpinen-4-ol, alpha-caryophyllene, and trans-longipinocarveol were the major compounds. In this study, both EOs were analyzed for anti-inflammatory potential on induced edema in rat ears, and the results showed that at doses of 50 µg/ear, they exhibited significant anti-inflammatory effect (*p* < 0.05), with inhibitions of up to 80%. According to the authors, these results were in accordance with the 100 *µ*g/ear hydrocortisone dose, which showed 54.8% of edema inhibition [118].

Six EOs from *Lamiaceae* family (*Perilla frutescens*, *Mentha haplocalyx*, *Pogostemon cablin*, *R. officinalis*, *Lavandula angustifólia,* and *Scutellaria baicalensis)* were studied regarding their anti-inflammatory potential. The major compounds found were linalool (0.05–46.55%), *α*-pinene (0.12–45.35%), *o*-cymene (0.91–41.20%), patchouli alcohol (28.27%), dl-menthol (21.12%), isobornyl acetate (22.52%), D-limonene (0.01–18.42%), *α*-terpineol (0.07–4.88%), and *ß*-pinene (0.08–2.03%). The anti-inflammatory tests were performed on the ears of rats of 6–8 weeks of age and bodyweight of 18 ± 2 g. They were induced by 12-O-tetradecanoyl phorbol-13-acetate (TPA), and the drug ibuprofen was used as positive control. All six essential oils exhibited anti-inflammatory activity, and the essential oils isolated from *P. cablin* remarkably inhibited the formation of ear edema (29.87–81.25% inhibition). Similarly, *R. officinalis* and *Scutellaria baicalensis* EOs worked better than ibuprofen (positive control) [135].

Thymol was the major compound found in *Thymus vulgaris* essential oil from two different regions of Algeria (Mostaganem-EO.TM and Tlemcen-EO.TT), with contents of 59.5% and 67.7%, respectively. The anti-inflammatory activity was evaluated *in vivo* based on the inhibition of paw edema induced by carrageenan injection. As a result, both EO samples showed anti-inflammatory activities after 6 hours of administration (400 mg/kg), reducing paw edema by 58.4% for EO.TT and 50.4% for EO.TM [136]. In the study by Avola et al. [137], *Origanum vulgare* EO presented as major compounds carvacrol (35.95–0.22%), thymol (25.2–0.27%), *p*-cymene (21.54–0.35%), and linalool (4.26–0.05%). This essential oil was tested to characterize the level of oxidative stress and evaluate the changes in intracellular ROS levels caused by IFN*γ* and histamine in the presence or absence of 25 *μ*g/mL of oil. Confluent NCTC 2544 cells were treated with H2DCFDA 72 h after stimulation. This ROS levels can cause inflammation-induced cellular damage. In this study, the results pointed out that cells pretreated with *O. vulgare* essential oil at 25 *μ*g/mL or indomethacin at 10 *μ*M significantly reduced IFN*γ*- and histamine-induced ROS production.

The chemical profile of *Stachys lavandulifolia* essential oil (EOSL) was characterized by the main compounds (-)-*α*-bisabolol (56.4%), bicyclogermacrene (5.3%), *δ*-cadinene (4.2%), and spathulenol (2.9%). And the anti-inflammatory activity of (-)-*α*-bisabolol (BIS) and EOSL (50 mg/kg) was evaluated using carrageenan-induced inflammatory response in rats (2% in 0.2 mL). The results showed that both EOSL and BIS possessed significant inhibitory effects (*p* < 0.05 or *p* < 0.01 or *p* < 0.001) on different orofacial pain tests, but BIS proved to be more effective, significantly reducing nociceptive behavior in all tests [138].

## 7. Antimicrobial Activity

### 7.1. Antibacterial Activity

*Lamiaceae* family has great importance in the economic scenario, especially in the gastronomic sector, in which they are used as culinary herbs. Thus, there has been an incessant search for new antimicrobial agents from the secondary metabolism of plants [8], which according to Nieto (2017) can increase the shelf life of food products [7].

Essential oils from aerial parts (leaves) of *Teucrium africanum* and *T. trifidum* were characterized by the sesquiterpene hydrocarbons *α*-cubebene and *ß*-cubebene, respectively. In this study, they were evaluated for their antimicrobial potential. *T. africanum* EO showed minimum inhibitory concentration (MIC) equal to 0.16 mg/mL against Gram-positive bacterium *Streptococcus pyogenes* (ATCC 25923). Similarly, *T. trifidum* EO demonstrated remarkable antimicrobial activity with the MIC of 2 mg/mL against Gram-positive bacterium *Staphylococcus aureus* (ATCC 8668) [139].

In another study, the essential oil from the leaves and flowers of *Origanum compactum*, collected in six regions of Morocco was characterized by the major compounds carvacrol (2.18–63.65%), *p*-cymene (6.69–42.64%), and thymol (0.16–42.37%). The antimicrobial activity of *O. compactum* EO was quite effective, being most active against *Escherichia coli*, *Listeria innocua*, and *Staphylococcus aureus* with inhibitory zones of 29.00 ± 0.35 mm, 49.00 ± 1.00 mm, and 43.00 ± 0.35 mm, respectively [140].

The major compounds such as citronellal (14.40%), isogeraniol (6.40%), and geranyl acetate (10.20%) characterized the leaf essential oil of *Melissa officinalis*. It showed significant antimicrobial activity against *Pseudomonas aeruginosa*, *Klebsiella pneumonia, Staphylococcus aureus,* and *Citrobacter koseri* when compared with the conventional antibiotics cefaclor, oxacillin, and vancomycin [141].

Khan et al. [142] evaluated the chemical composition of the leaf essential oil of *O. vulgare*, which presented carvacrol (70.2 ± 1.37%) and *γ*-terpinene (5.6 ± 0.11%). In this study*, O. vulgare* EO was evaluated for its antimicrobial potential against Gram-positive (*Micrococcus luteus* and *Staphylococcus aureus*) and Gram-negative (*Escherichia coli* and *Pseudomonas aeruginosa*) bacteria in comparison with its purified compound carvacrol. The results showed that carvacrol was more effective and completely inhibited the growth of *E. coli* at 200 mg/mL and also retarded the growth of *P. aeruginosa*, with IC_50_ value of 151 mg/mL. The essential oil, on the other hand, inhibited bacterial growth at concentrations of 270, 263, 214, and 383 mg/mL for *M. luteus*, *S. aureus, E. coli,* and *P. aeruginosa,* respectively.

In the study conducted by Niksic et al. [119], major compounds carvone (56,4%), limonene (16,2%), 1,8-cineole (7%), *ß*-pinene (2,4), and *α*-terpinene (2,3%) characterized Mentha spicata essential oil. It exhibited significant bactericidal activity against both Gram-positive and Gram-negative microorganisms, with M. spicata essential oil being more sensitive and showing greater zone of inhibition against *Escherichia coli* (11.8–21 mm), *Salmonella enterica* (8–18 mm), and *Pseudomonas aeruginosa* (10–16 mm). Gram-positive bacteria, on the other hand, showed moderate antimicrobial activity at concentrations of 1%, 5%, and 10%, against *S. aureus* (8–13 mm), *Staphylococcus* epidermidis (10.1–11.2 mm), and *Bacillus* subtilis (9–11.5 mm). According to the authors, Mentha spicata antibacterial activity can be attributed to the presence of several chemical groups, such as oxygenated monoterpenes and hydrocarbons, which favors the use of M. spicata essential oil as an antiseptic agent in the pharmaceutical and food industries.

The essential oils of four species of the genus Thymus (*T. vulgaris*, T. zygis, T. serpyllum, and T. pulegioides) were analyzed, and their chemical profile was characterized by oxygenated monoterpenes and thymol, which was the major chemical constituent. It presented the following contents: 37.7%, 41.7%, 13.7%, and 44.5% for *T. vulgaris*, *T. zygis, T. serpyllum,* and *T. pulegioides*, respectively. The determination of their antibacterial activity against the Gram-positive bacterium *Streptococcus mutans* was performed by turbidity measurement, determination of colony-forming units (CFUs), and the live/dead staining method. In the turbidity test, essential oils of *T. zygis* and *T. Pulegioides* had the highest minimum inhibitory concentration (MIC equal to 0.5 mg/mL), followed by *T. vulgaris* (MIC = 0.75 mg/mL) and *T. serpyllum* (MIC = 0.9 mg/mL). Regarding CFU, all four essential oils significantly affected S. *mutans* growth. The lowest CFU value was found for T. serpyllum (1,750 CFU [bacterial/ml]), followed by *T. vulgaris* (3,500 CFU [bacterial/ml]), *T. zygis* (4,500 CFU [bacterial/ml]), and *T. pulegioides* (27,500 CFU [bacterial/ml]). Regarding the live/dead staining method, *T. vulgaris* essential oil had the strongest *in vitro* antimicrobial activity against S. *mutans*, followed by *T. pulegioides* and *T. serpyllum.* In contrast, the essential oil of *T. zygis* had the weakest effect [143].

**Figure 3:**
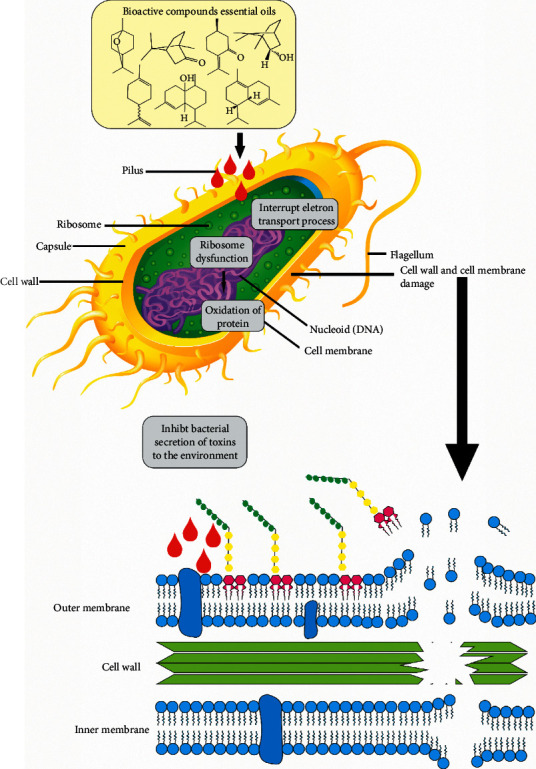
Potential mechanism of action of essential oils on bacteria, adapted from [145,146].

Leaf essential oil of *Salvia ringens* was characterized by 1.8-cineole (31.99%), camphene (17.06%), borneol (11.94%), and *α*-pinene (11.52%). It was tested against six Gram-negative bacteria: *E. coli* (ATCC25922*), Salmonella typhimurium* (ATCC14028), *Salmonella enteritidis* (ATCC13076), *Pseudomonas tolaasii* (NCTC387), *Pseudomonas aeruginosa* (ATCC27853), and *Proteus mirabilis* (ATCC14273), and five Gram-positive bacteria: *Staphylococcus aureus* (ATCC25923), *Bacillus cereus* (ATCC10876), *Micrococcus flavus* (ATCC14452), *Sarcina lutea* (ATCC10054), and *Listeria monocytogenes* (ATCC15313). The results showed that *S. ringens* EO showed the strongest antibacterial activity with MIC equal to 9.50–17.10 mg/mL [144]. In Table 3, results of the antibacterial activity of *Lamiaceae* essential oils are shown.  Figure 3 shows a probable mechanism of action of essential oils in bacteria.

### 7.2. Antifungal Activity

Fungal infections can be very dangerous for humans, especially when it concerns food, because fungi have the ability to produce mycotoxins and also reduce or destroy the nutritional value of grains during storage. Thus, it is important to mention the numerous studies with *Lamiaceae* essential oils with antimicrobial properties against fungi [7]. EOs of *O. vulgare*, *Thymus capitatos,* and *Satureja thymbra* were analyzed and showed the following major constituents: carvacrol (82.48%), p-cymene (5.00%), and *γ*-terpinene (2.62%). They were tested against two phytopathogenic fungi (*Aspergillus Niger* and *Penicillium* spp.) isolated from slices of bread left outdoors at room temperature. Results showed that the addition of essential oils had significant effect (*p* < 0.05) on decreasing their colony surface area. Thus, oregano (*O. vulgare*), thyme (*Thymus capitatus*), and pink savory (*S. thymbra*) can be incorporated into bread recipes and be used in the food industry, as they have antimicrobial properties [147].

In the study by Niksic et al. [148], *Lepechinia mutica* EO was characterized by shyobunol (10.80%), 3-carene (8.69%), *δ*-cadinene (6.96%), and globulol (5.91%), and it was tested against three serious human pathogenic fungi: *Candida albicans*, *Trichophyton rubrum,* and *Microsporum canis*, and two potent plant pathogens: *Pyricularia oryzae* and *Fusarium graminearum*. Compared with the positive controls amphotericin B and voriconazole, *L. mutica* EO exhibited moderate activity against *M. canis* and *T. rubrum*, having MIC values ranging from 2.2 to 4.5 mg/mL.

Rus et al. [149] evaluated the chemical composition of EOs from three species of the *Lamiaceae* family (*T. vulgaris, T. serpyllum,* and *Satureja montana*), which showed the following major compounds: p-cymene, *γ*-terpinene, and carvacrol. Their antifungal activity was evaluated against *Verticillium dahliae* and *Penicillium aurantiogriseum* at concentrations ranging from 0.25–15 mg/L. The essential oils of *T. vulgaris, T. serpyllum,* and *Satureja montana* exhibited mycelial growth inhibition (MGI) equal to 10%, 30%, and 18%, respectively, against *V. dahlia,* and 0%, 99%, and 37% against *P. aurantiogriseum* at 0.25 mg/L. At the other concentrations, growth was almost nonexisting. These results show that *T. vulgaris* EO was the most effective, especially against *P. aurantiogriseum*, which was highly sensitive.

The major compounds linalool (48.4%), 1,8-cineole (12.2%), eugenol (6.6%), methyl cinnamate (6.2%), *α*-cubebene (5.7%), caryophyllene (2.5%), *ß*-ocimene (2.1%), and *α*-farnesene (2.0%) characterized the chemical profile of the essential oil from *O. basilicum* leaves. It was tested against *Aspergillus flavus* at concentrations of 500, 750, and 1000 ppm, and the results showed that at 500 ppm, this EO showed inhibition rate of 30%; at 750 ppm, 50%; and at 1000 ppm, 70%. These results are promising for curing mycotic infections and as a pharmaceutical preservative against *A. flavus* growth. It may also be used for aflatoxin B1 production [150].

**Figure 4:**
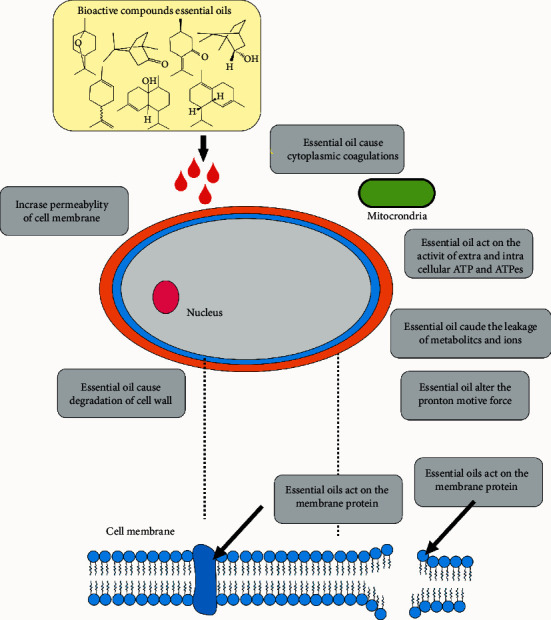
Potential mechanism of action of essential oils in fungi, adapted from [152].

*Ocimum tenuiflorum* essential oil was characterized by methyl eugenol (84.7%) and *ß*-caryophyllene (7.4%), whereas *O. basilicum* EO had its chemical profile characterized by the major constituents linalool (35.1%), eugenol (20.7%), and 1,8-cineole (9.9%). In this study, they were tested against *C. albicans, C. tropicalis, C. krusei, C. guilliermondii, C. parapsilosis, Cryptococcus neoformans, Trichophyton mentagrophytes, T. mentagrophytes var. interdigitale, Trichophyton rubrum, T. verrucosum, Microsporum canis, M. gypseum, Epidermophyton floccosum, Aspergillus niger, A. fumigatus,* and *A. flavus*, with significant results. The essential oil of *O. tenuiflorum* exhibited prominent antifungal activity against *C. neoformans* (MIC = 0.16 *μ*L/mL) and dermatophyte fungi (0.32 *μ*L/mL). However, it had no fungicidal effect against *Aspergillus niger* (MLC> 10 *μ*L/mL), while *O. basilicum* EO hindered the development of this kind of fungus, presenting fungicidal activity (MLC = 2.5–5 *μ*L/mL) and MIC equal to 0.64–1.25 *μ*L/mL [151]. In Table 4, the antifungal activity of *Lamiaceae* essential oils is shown.  Figure 4 shows the possible mechanism of action of essential oils on fungi.

## 8. Conclusion

*Lamiaceae* species and, consequently, their essential oils may have peculiarities according to their cultivation system, climate, and location. Thus, some chemical and biological properties tend to change, showing great potential, especially regarding medicinal applications.

They have been used to fight certain diseases due to their antioxidant, antifungal, antibacterial, and anti-inflammatory actions. Additionally, there are other *Lamiaceae* species that act against insects, as well as on environmental remediation (phytoremediation) and thermal protection (green roofs).

Because this botanical family is extremely versatile, more studies on its compounds must be conducted since it has great pharmacological potential, with a promising future. Therefore, this review contributes to future studies on *Lamiaceae* and encourages the use of alternative natural resources for different purposes.

## Figures and Tables

**Table 1:** Chemical composition and yield of essential oils from *Lamiaceae* species.

Species	Plant part	Essential oil yield	Main compounds	Reference
*Aeollanthus suaveolens* Mart. ex Spreng	Leaves	1.6%	Massoia lactone, linalool,(*E*)-B-farnesene, geraniol, 2,5-dimethoxy-p-cymene	[95]
*Calamintha nepeta* (L.) Kuntze	Leaves	∗	Pulegone, menthone, limonene	[96]
*Clinopodium macrostemum* (Moc. and Sessé ex Benth.) Kuntze	Leaves	0.80%	linalool, nerol, caryophyllene, menthone, geranyl acetate, terpineol, pulegone	[97]
*Hyptis dilatata* Benth.	Leaves	∗	Fenchone, 3-carene, *α*-pinene, *ß*-caryophyllene, limonene, *ß*-pinene, and camphor	[98]
*Hyptis martiusii* Benth.	Leaves	0.34%	1,8-cineole, d-carene, camphor, limonene, germacrene B	[99]
*Lavandula dentata* L.	Leaves and stems	∗	1,8-cineole, isolimonene, thuj-3-en-10-al, trans-pinocarveol	[100]
*Melissa officinalis* L.	Leaves and flowers	0.10%	Citral, caryophyllene oxide, citronellal, geraniol, geranyl acetate, *ß*-caryophyllene	[101]
*Mentha arvensis* L.	Leaves and flowers	∗	Citronellal and nerol	[102]
*M. piperita* L	Leaves and flowers	∗	Menthone, menthol, pulegone and menthyl acetate	[103]
*Minthostachys mollis* (Benth.) Griseb.	Leaves	0.98%	Menthone, pulegone, cis-dihydrocarvone, carvacryl acetate, linalyl acetate, and linalool	[104]
*Ocimum basilicum* L.	Leaves	1.56 ± 0.15%	linalyl acetate and linalool	[105]
*O. gratissimum* L.	Leaves	∗	1,8-Cineole, eugenol, 4-terpineol	[91]
*O. gratissimum*	Leaves and flowers	∗	Thymol, eugenol, 1,8-cineole, E-caryophyllene, *ß*-selinene	[106]
*Origanum scabrum* Boiss. and Heldr.	Leaves	1.5%	Carvacrol, thymol, p-cymene,*γ*-terpinene	[107]
*Origanum vulgare* L.	Leaves	∗	4-terpineol, sabinene hydrate, thymol	[92]
*Plectranthus amboinicus* (Lour.) Spreng.	Leaves	0.009%	Thymol, *ß*-pinene, *γ*-terpinene, caryophyllene	[108]
*Plectranthus barbatus var. grandis* (L.H. Cramer) Lukhoba and A.J. Paton	Leaves	∗	*β*-caryophyllene, *α*-copaene, germacrene	[109]

**Table 2:** Antioxidant activity of essential oils from *Lamiaceae* species.

Species and plant part	Technique	Results	Reference
*Cedronella canariensis* (aerial parts)	DPPH	IC_50_ = 615.5 ± 76.5 *μ*g/mL	[114]
	ABTS	IC_50_ = 10.5 ± 0.6 *μ*g/mL	
	FRAP	IC_50_ = 3.8 ± 1.4 6 *μ*mol TE/g	
*Mentha piperita* (leaves)	DPPH	Radical scavenging = 92.6 ± 6.86%	[115]
	Reducing power	Reducing power = 0.9 ± 0.3	
*M. pulegium* (aerial parts)	DPPH	IC_50_ = 321.41 ± 2.53 *μ*g/mL	[116]
	FRAP	IC_50_ = 58.27 ± 2.72 *μ*g/mL	
*M. rotundifolia* (leaves)	DPPH	IC_50_ = 2222.2 ± 25.2 *μ*g/mL	[117]
	ABTS	IC_50_ = 133.8 ± 4.8 *μ*g/mL	
	Reducing power	IC_50_ = 166.6 ± 1.9 *μ*g/mL	
	Phosphomolybdate	IC_50_ = 45.2 ± 1.2 *μ*g/mL	
*M. spicata* (aerial parts)	DPPH	IC_50_ = 3450 ± 172.5 *μ*g/mL	[118]
	ABTS	IC_50_ = 40.2 ± 0.2 *μ*g/mL	
	FRAP	IC_50_ = 215 ± 4.50 *μ*g/mL	
*M. spicata* (leaves)	DPPH	IC_50_ = 41, 23 *µ*g/mL	[119]
*O. basilicum* (aerial parts)	DPPH	IC_50_ = 4.04 ± 0.09–0.21 ± 0.02 mg/mL	[120]
	*β*-carotene	Bleaching = 23.8 ± 0.6–85.3 ± 1.0%	
*Origanum dictamnus* (flowers)	DPPH	IC_50_ = 0.0459 ± 0.0042% (v/v)	[121]
*O. floribundum* (aerial parts)	DPPH	IC_50_ = 369.9 ± 3.1– 1091.7 ± 4.5 *µ*g/mL	[122]
	Reducing power	IC_50_ = 230 ± 5.2– 315 ± 3.9 *µ*g/mL	
	ABTS	IC_50_ = 33.6 ± 0.3– 95.5 ± 2.2 *µ*g/mL	
*O. vulgare* (aerial parts)	ABTS	IC_50_ = 14,00257 mg/mL	[123]
*O. vulgare* (flowers)	DPPH	EC_50_ = 0.68 mL/mL	[124]
*O. vulgare* (stems)	DPPH	EC_50_ = 1.82 mL/mL	[124]
*R. officinalis* (aerial parts)	DPPH	IC_50_ = 523.41 ± 8.25 *µ*g/mL	[116]
	FRAP	IC_50_ = 85.74 ± 7.57 *µ*g/mL	
*R. officinalis* (aerial parts)	DPPH	IC_50_ = 10.08 ± 0.15 *µ*g/mL	[125]
	TBARS	IC_50_ = 1.76 ± 0.02 *µ*g/mL	
*R. officinalis* (aerial parts)	DPPH	IC_50_ = 13.00 ± 0.51 *µ*g/mL	[126]
*Satureja hortensis* (aerial parts)	DPPH	IC_50_ = 13.45 ± 0.35 *µ*g/mL	[127]
*Scutellaria immaculate* (aerial parts)	DPPH	IC_50_ = 82.8 ± 3.1 *µ*g/mL	[128]
	ABTS	IC_50_ = 37.8 ± 0.9 *µ*g/mL	
	FRAP	IC_50_ = 720.19 ± 4.8 *µ*g/mL	
*S. ramosissima* (aerial parts)	DPPH	IC_50_ = 82.8 ± 3.1 *µ*g/mL	[128]
	ABTS	IC_50_ = 93.6 ± 0.8 µg/mL	
	FRAP	IC_50_ = 837.23 ± 3.2 *µ*g/mL	
*S. schachristanica* (aerial parts)	DPPH	IC_50_ = 57.6 ± 2.7 *µ*g/mL	[128]
	ABTS	IC_50_ = 66.6 ± 1.2 *µ*g/mL	
	FRAP	IC_50_ = 779.64 ± 8.6 *µ*g/mL	
*Teucrium flavum* (aerial parts)	DPPH	IC_50_ = 31.5 ± 1.8 *µ*g/mL	[129]
*Thymus capitatus* (Leaves)	DPPH	IC_50_ = 0.619 ± 0.11 *µ*g/mL	[130]
	FRAP	IC_50_ = 2,13 ± 0.07 *µ*g/mL	
	TAC	IC_50_ = 0.78 ± 0.14 *µ*g/mL	

**Table 3:** Antibacterial activity of *Lamiaceae* essential oils.

Species	Bacteria	Method applied	Results	Reference
*Mentha spicata*	*E. coli*	Disc-diffusion	11.8–21 mm	[119]
	*S. enterica*		8–18 mm	
	*P. aeruginosa*		10–16 mm	
	*S. aureus*		8–13 mm	
	*S. epidermidis*		10.1–11.2 mm	
	*B. subtilis*		9–11.5 mm	
*Melissa officinalis*	*P. aeruginosa*	Agar-disc-diffusion	16.0 ± 1.2 mm	[141]
	*K. pneumonia*		3.0 ± 0.6 mm	
	*S. aureus*		20.0 ± 1.6 mm	
	*C. koseri*		14.0 ± 1.0 mm	
*Origanum compactum*	*E. coli* K12	Microdilution	29.00 ± 0.35 mm	[140]
	*L. innocua* 4030		49.00 ± 1.00 mm	
	*S. aureus* 25.923		43.00 ± 0,35 mm	
*O. vulgare*	*M. luteus*	Microdilution	*270 mg/mL*	[142]
	*S. aureus*		*263 mg/mL*	
	*E. coli*		*214 mg/mL*	
	*P. aeruginosa*		*383 mg/mL*	
*Salvia ringens*	*E. coli*	Microdilution	14.25	[144]
	*S. typhimurium*		14.25	
	*S. enteritidis*		11.40	
	*P. tolaasii*		14.25	
	*P. aeruginosa*		17.10	
	*P. mirabilis*		17.10	
	*S. aureus*		9.50	
	*B. cereus*		9.50	
	*M. flavus*		9.50	
	*S. lutea*		11.40	
	*L. monocytogenes*		9.50	
*Teucrium africanum*	*S. pyogenes* (ATCC)	Microdilution	0.16 mg/mL	[139]
*T. trifidum*	*S. aureus*	Microdilution	2 mg/mL	
*Thymus pulegioides*	.	Turbidity measurements	0.5 mg/mL	[143]
		CFU	27.500 bacterial/mL	
T. serpyllum	S. mutans	Turbidity measurements	0.9 mg/mL	
		CFU	1.750.000 bacterial/mL	
T. vulgaris	S. mutans	Turbidity measurements	0.75 mg/mL	
		CFU	3500 bacterial/mL	
T. zygis	S. mutans	Turbidity measurements	0.5 mg/mL	
		CFU	4500 bacterial/mL	

**Table 4:** Antifungal activity of *Lamiaceae* essential oils.

Species	Fungi	Method applied	Results	Reference
*Lepechinia mutica*	*C. albicans*	Broth microdilution	MIC >9 mg/mL	[148]
	*M. canis*		2.2 < MIC ≤4.5 mg/mL	
	*T. rubrum*		2.2 < MIC ≤4.5 mg/mL	
	*F.graminearum*		MIC >9 mg/mL	
	*P.oryzae*		MIC >9 mg/mL	
*O. basilicum*	*A. flavus*	Potato dextrose agar (PDA)	500 ppm: 30% 750 pp : 50% 1000 ppm: 70%	[150]
*O. basilicum*	*C. albicans*	Sabouraud dextrose agar (SDA)	MIC: 1.25 *μ*L/mL MLC: 2.5 *μ*L/mL	[151]
	*C. tropicalis*		MIC: 2.5–1.25 *μ*L/mL MLC: 2.5 *μ*L/mL	
	*C. krusei*		MIC: 1.25 *μ*L/mL MLC: 2.5 *μ*L/mL	
	*C. guilliermondii*		MIC: 1.25 *μ*L/mL MLC: 1.25 *μ*L/mL	
	*C. parapsilosis*		MIC: 1.25 *μ*L/mL MLC: 2.5 *μ*L/mL	
	*C. neoformans*		MIC: 0.16–0.32 *μ*L/mL MLC: 0.64–0.32 *μ*L/mL	
	*T. mentagrophytes*		MIC: 0.64 *μ*L/mL MLC: 1.25 *μ*L/mL	
	*T. mentagrophytes var. interdigitale*		MIC: 0.64–0.32 *μ*L/mL MLC: 1.25 *μ*L/mL	
	*T. rubrum*		MIC: 0.64 *μ*L/mL MLC: 1.25 *μ*L/mL	
	*T. verrucosum*		MIC: 0.64 *μ*L/mL MLC: 1.25 *μ*L/mL	
	*M. canis*		MIC: 0.64 *μ*L/mL MLC: 1.25 *μ*L/mL	
	*M. gypseum*		MIC: 0.64 *μ*L/mL MLC: 1.25 *μ*L/mL	
	*E. floccosum*		MIC: 0.64 *μ*L/mL MLC: 0.64 *μ*L/mL	
	*A. niger*		MIC: 0.64 *μ*L/mL MLC: 5 *μ*L/mL	
	*A. fumigatus*		MIC: 1.25 *μ*L/mL MLC: 5 *μ*L/mL	
	*A. flavus*		MIC: 1.25 *μ*L/mL MLC: 2.5 *μ*L/mL	
*O. tenuiflorum*	*C. albicans*	Sabouraud dextrose agar (SDA)	MIC: 0.64 *μ*L/mL MLC: 1.25 *μ*L/mL	
	*C. tropicalis*		MIC: 0.64 *μ*L/mL MLC: 1.25 *μ*L/mL	
	*C. krusei*		MIC: 0.64 *μ*L/mL MLC: 2.5 *μ*L/mL	
	*C. guilliermondii*		MIC: 0.64 *μ*L/mL MLC: 1.25 *μ*L/mL	
	*C. parapsilosis*		MIC: 0.64 *μ*L/mL MLC: 2.5 *μ*L/mL	
	*C. neoformans*		MIC: 0.16 *μ*L/mL MLC: 0.64 *μ*L/mL	
	*T. mentagrophytes*		MIC: 0.32 *μ*L/mL MLC: 0.32 *μ*L/mL	
	*T. mentagrophytes var. interdigitale*		MIC: 0.32 *μ*L/mL MLC: 0.64 *μ*L/mL	
	*T. rubrum*		MIC: 0.32 *μ*L/mL MLC: 0.64 *μ*L/mL	
	*T. verrucosum*		MIC: 0.32 *μ*L/mL MLC: 0.64 *μ*L/mL	
	*M. canis*		MIC: 0.32 *μ*L/mL MLC: 0.64 *μ*L/mL	
	*M. gypseum*		MIC: 0.32 *μ*L/mL MLC: 0.32–0.64 *μ*L/mL	
	*E. floccosum*		MIC: 0.32 *μ*L/mL MLC: 0.32 *μ*L/mL	
	*A. niger*		MIC: 0.64 *μ*L/mL MLC: > 10 *μ*L/mL	
	*A. fumigatus*		MIC: 0.64 *μ*L/mL MLC: > 10 *μ*L/mL	
	*A. flavus*		MIC: 0.64 *μ*L/mL MLC: > 10 *μ*L/mL	
*Origanum vulgare*	*Penicillium*	Potato dextrose agar (PDA)	0 *μ*L–3 cm^2^ 12.5 *μ*L–2 cm^2^ 25 *μ*L–2 cm^2^ 50 *μ*L–1 cm^2^	[147]
	*A. niger*		0 *μ*L–3 cm^2^ 12.5 *μ*L–2 cm^2^ 25 *μ*L–2 cm^2^ 50 *μ*L–1 cm^2^	
*Satureja thymbra*	*Penicillium*		0 *μ*L–4 cm^2^ 12.5 *μ*L–3 cm^2^ 25 *μ*L–2cm^2^ 50 *μ*L–1 cm^2^	
	*A. niger*		0 *μ*L–3 cm^2^ 12.5 *μ*L–3 cm^2^ 25 *μ*L–2 cm^2^ 50 *μ*L–1 cm^2^	
*S. montana*	*V. dahliae*	CYGA (chloramphenicol-yeast-glucose-agar)	0.25 mg/L–18%	[149]
	*Pe. aurantiogriseum*		0.25 mg/L–37%	
*Thymus capitatus*	*Penicillium*	Potato dextrose agar (PDA)	0 *μ*L–4 cm^2^ 12.5 *μ*L–3 cm^2^ 25 *μ*L–2 cm^2^ 50 *μ*L–1 cm^2^	[147]
	*A. niger*		0 *μ*L–3 cm^2^ 12.5 *μ*L–3 cm^2^ 25 *μ*L–2 cm^2^ 50 *μ*L–1 cm^2^	
*T. vulgaris*	*V. dahliae*	CYGA (chloramphenicol-yeast-glucose-agar)	0.25 mg/L–10%	[149]
	*P. aurantiogriseum*		∗∗∗∗	
*T. serpyllum*	*V. dahliae*	CYGA (chloramphenicol-yeast-glucose-agar)	0.25 mg/L–30%	[149]
	*P. aurantiogriseum*		0.25 mg/L–99%	

## Data Availability

The datasets generated and analyzed during the current study are available in the databases, such as PubMed, Google Scholar, Web of Science, Scopus, and Science Direct (datasets can be requested from the corresponding author upon formal request).
